# Health professionals’ perspectives on breast cancer risk stratification: understanding evaluation of risk versus screening for disease

**DOI:** 10.1186/s40985-019-0111-5

**Published:** 2019-02-28

**Authors:** Svetlana Puzhko, Justin Gagnon, Jacques Simard, Bartha Maria Knoppers, Sophia Siedlikowski, Gillian Bartlett

**Affiliations:** 1 Department of Family Medicine, Faculty of Medicine, McGill University, 5858 Chemin de la Côte-des-Neiges, Suite 300, Montréal, Québec H3S 1Z1 Canada; 2 Genomics Center, CHU de Québec-Université Laval Research Center, Room R4-4787, 2705 Laurier Blvd, Québec, Québec G1V 4G2 Canada; 3 Genome Quebec Innovation Centre of Genomics and Policy, Department of Human Genetics, Faculty of Medicine, McGill University, 3640 University Street, Room W-315, 740 Dr. Penfield Ave, 5214, Montréal, Québec H3A 0C7OG1 Canada; 4 Department of Molecular Medicine, Faculty of Medicine, Université Laval, Québec, Canada

**Keywords:** Breast cancer screening, Deliberative stakeholder consultations, Risk stratification, Personalized screening, Program implementation

## Abstract

**Background:**

Younger women at higher-than-population-average risk for breast cancer may benefit from starting screening earlier than presently recommended by the guidelines. The Personalized Risk Stratification for Prevention and Early Detection of Breast Cancer (PERSPECTIVE) approach aims to improve the prevention of breast cancer through differential screening recommendations based on a personal risk estimate. In our study, we used deliberative stakeholder consultations to engage health professionals in an in-depth dialog to explore the feasibility of the proposed implementation strategies for this new personalized breast cancer screening approach.

**Methods:**

Deliberative stakeholder consultation is a qualitative descriptive study design used to engage health professionals in the discussion, while the mediators play a more passive role. A purposeful sample of 11 health professionals (family physicians and genetic counselors) working in Montreal was used. The deliberations were organized in two phases, including small group deliberations according to the deliberants’ health profession and a mixed group deliberation combining participants from the small groups. Inductive thematic content analysis was performed on the transcripts by two coders to create the deliberative and analytic outputs. Quality of deliberations was assessed quantitatively using the de Vries method and qualitatively using participant observation.

**Results:**

One of our key findings was that health professionals lacked understanding of the two steps of the screening approach: risk stratification “screening,” which is an evaluation for the level of risk and screening for disease. As part of this confusion, the main topic of concern was a justification of program implementation as a population-wide screening, based on their uncertainty that it will be beneficial for women with near-population risks. Despite the noted difficulties concerning implementation, health professionals acknowledged the substantial benefits of the proposed PERSPECTIVE program.

**Conclusions:**

Our study was the first to evaluate the perspectives of health professionals on the implementation and benefits of a new program for breast cancer risk stratification with the purpose of personalizing screening for disease. This new multi-step approach to screening requires more clarity in communication with health professionals. To implement and maintain effective screening, engagement of family physicians with other health professionals or even development of a centralized public health system may be needed.

## Background

Breast cancer is the most common cancer in women and the second most frequently diagnosed malignant tumor worldwide, with an incidence of about 12% [1–4]. It comprises 25.2% of all newly diagnosed cancers [1, 4]. During recent years, survival rates for breast cancer in developed countries have improved due to detection at earlier stages and development of new treatment strategies [4]. Breast cancer, however, remains the fifth major cause of death from cancer among women worldwide [4]. In Canada, the lifetime risk of developing breast cancer was 12.4% in 2017 [5]. Due to increased mammography screening, along with the use of more effective therapies after breast cancer surgery, the breast cancer mortality rate in Canada has been declining since the mid-1980s [6–8]. Nevertheless, more than 26,300 new cases of breast cancer are diagnosed annually in Canada, and over 5000 women die of breast cancer every year.

Breast cancer-related morbidity and mortality can be decreased with guideline-recommended population-wide screening [9, 10]. In Canada, participation in mammography screening programs was associated with 40% reduction in breast cancer mortality (95% CI 33–48%) [11]. Moreover, breast cancer treatment (chemotherapy, radiation therapy, and surgery) is more effective when breast cancer is detected earlier [11]. Presently, however, the Canadian Task Force on Preventive Health Care (CTFPHC) only recommends starting regular biennial mammography screening for women at the age of 50 [12–14] even though breast cancer in younger women is often more aggressive [15]. The CTFPHC recommendations are based on a synthesis of the available evidence on the balance of benefits and harms. The harms comprise over-diagnosis, false-negative results leading to false reassurance, overtreatment, and unnecessary costs. Even though these issues may present serious disadvantages of early screening for some women, others may benefit from starting screening before the age of 50. In fact, there is still considerable debate on whether younger women would benefit from routine screening [16]. Notably, the CTFPHC recommendations are developed for women with the population-average risk for breast cancer and do not take into account that women may have different levels of risk for this disease and, therefore, may need a personalized approach to screening. Benefits of breast cancer screening for younger women with a higher-than-population-average level of risk have been shown in several recent studies [17, 18]. Moreover, offering annual mammography screening to younger women at high risk of developing breast cancer is recommended in the UK National Institute of Health and Clinical Excellence (NICE) [19] as well as by Canadian experts [20]. In fact, during recent decades, a more personalized approach to screening and prevention of cancer has become possible due to the development of risk prediction models [21, 22]. These models calculate individual risk estimates for the potential development of cancer based on a woman’s personal characteristics, environmental risk factors, and information from genetic testing.

**Fig. 1:**
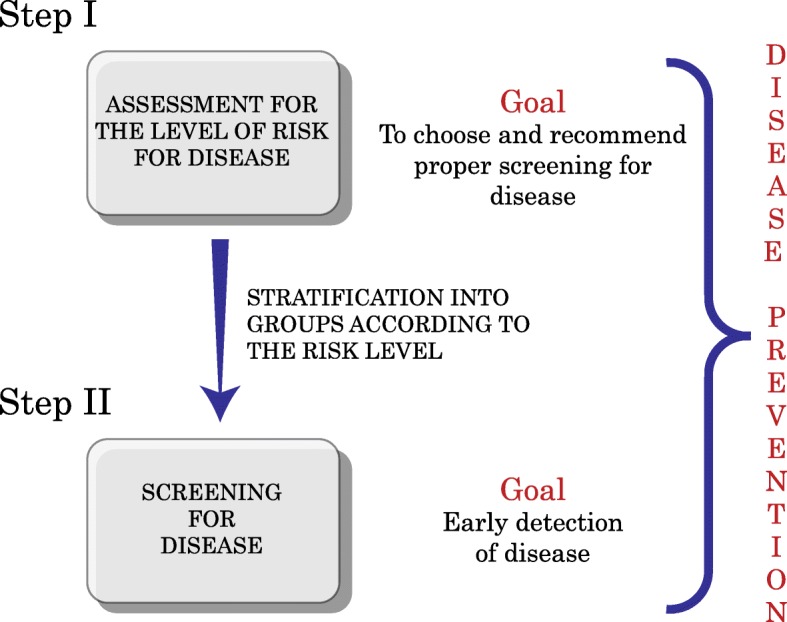
Stepwise approach to screening

This approach is exemplified in the PERSPECTIVE project (Personalized Risk Stratification for Prevention and Early Detection of Breast Cancer). The goal of PERSPECTIVE is to improve the detection and prevention of breast cancer through differential screening recommendations based on a personal risk estimate for each woman [20]. Implementation of this program would bring breast cancer screening to a new, more sophisticated, level of disease prevention as screening for breast cancer will then become a stepwise procedure (Fig. 1).

Step I is the evaluation of a woman’s individual level of risk of developing breast cancer. To estimate individual risk, data on personal characteristics and medical and family history are used, as well as information on breast density and genomic profiling test if available. For individual risk estimation, the Breast and Ovarian Analysis of Disease Incidence and Carrier Estimation Algorithm (BOADICEA) risk prediction model is used. BOADICEA is a free online tool designed by the Centre for Cancer Genetic Epidemiology at the University of Cambridge (UK). It is applied to calculate 10-year and lifetime age-specific risks of breast cancer and probabilities to be a mutation carrier [22]. One of the advantages of BOADICEA is its flexibility: the model allows calculations of risk by integration of known risk factors in the absence of unknown ones. For example, if data on breast density and genomic profiling test are unavailable, including when these tests have not been conducted, medical and family history are sufficient to calculate the risks. When one or both of these risk factors are included, however, the precision of the estimate is increased.

The goal of a personalized risk stratification approach is to assign a specific risk group to a woman based on her individual risk in order to provide recommendations for appropriate screening for early detection and prevention of disease tailored to this risk group. For example, this approach is meant to identify younger women (35–49 years) at higher risk of breast cancer, who will be recommended to follow a disease screening program specific for their risk level, including starting mammography earlier than at the age 50, which currently falls outside the standard screening recommendations. Upon completion of the first step of this approach, all women are expected to be given individualized recommendations to start step II of screening: screening for disease. The goal of this second step is early detection of disease and disease prevention. Implementation of the additional step (risk stratification) will allow women who are at higher-than-population risk to start appropriate follow-up earlier. This would increase the chances of detecting the disease at earlier, treatable stages. Women in the population-level risk group would follow the general population screening recommendations. Stratification, therefore, is expected to allow younger women with near-population risk who have a fear of a breast cancer diagnosis based on personal experiences (e.g., breast cancer in a close friend) to avoid unnecessary referrals and additional interventions and to allocate resources to those who would most benefit from them. Implementation of the risk stratification program is expected to help physicians and women make optimal decisions regarding screening for disease and, ultimately, improve early detection of breast cancer and further diminish disease-related morbidity and mortality.

The suggested breast cancer risk stratification approach transforms breast cancer screening into a more sophisticated, multi-step process. The implementation is expected to involve complex issues (i.e., use of genomics in the general population) and require prioritization of limited resources (i.e., population information versus individual consultation). Therefore, to assess the acceptability of implementation strategies in the clinical context, the involvement of health care professionals is needed. Even though several studies have been recently conducted to evaluate the interest and attitude of a general population and health professionals regarding a risk-stratified breast cancer screening approach [23–27], no studies, to our knowledge, was performed to involve health professionals in a dialog to discuss the implementation of this approach. In our study, we used deliberative stakeholder consultations to engage health care professionals in a meaningful exchange and generate an in-depth dialog to explore the feasibility of any proposed implementation strategies for targeted breast cancer screening and to optimize communication tools for the risk stratification model [28]. The two groups of health professionals, family physicians and genetic counselors, were chosen for the following reasons. Currently, in Canada, women enter into the breast cancer screening programs through a personal letter of invitation that they receive at the age of 50, through physician referral or through self-referral. Where organized screening programs are not available, screening services may be provided opportunistically through a primary care provider. Mammography screening results are disclosed to the woman and to her family physician; most often, it is the family physician who communicates the results to women and then coordinates follow-up. Genetic counselors are another group of health professionals primarily involved in communication with patients regarding their breast cancer risks. They deal with women with perceived breast cancer who have objective (e.g., strong family history) and subjective reasons to be concerned.

## Methods

Deliberative stakeholder consultation is a qualitative descriptive study design that uses a deliberative democratic approach to engage health professionals in the discussion, while the researchers and mediators play a more passive role [28]. The aim is to frame an in-depth discussion between expert stakeholders and examine the outcomes of the debate as well as the process that led to those outcomes. This method helps enrich the interpretation of research findings through the integration of various stakeholder viewpoints and perceptions, enhance the empowerment of stakeholders, and increase the probability of successful policy adoption [28]. The concept of deliberative democracy is based on the foundations of political equality and deliberation. Therefore, during the deliberations, we have ensured that the views of participants are duly considered by all stakeholders and that their expression is devoid of undue pressure or coercion.

A purposeful sample of participants was recruited consisting of 11 health professionals, working in Montreal and speaking French or English. Participants included family physicians recruited from academic units, community health centers (*Centre Local de Services Communautaires*—CLSC), and genetic counselors, both working in the Montreal region. Participants were recruited by telephone call or by email. The primary health care professionals (PCPs) were selected from the McGill University academic family medicine units as well as the McGill Practice-Based Research Network (PBRN) in order to ensure a relevant primary care community perspective.

The deliberative stakeholder consultations were organized in two phases. The first phase involved small group deliberations, restricted according to the deliberants’ health profession to minimize group polarization and provide an opportunity for each group to develop a collective position. The second phase involved a mixed group deliberation, combining participants from the small groups. In the first phase, three small group deliberations were held: one with genetic counselors and the other two with primary health care providers. Three to five deliberants participated in each discussion. They were conducted in a non-clinical setting, at the Family Medicine Department of McGill University. Two trained experts (GB and a genetic counselor) facilitated the session, and two note takers (JG and SP) were present during the deliberations. At least one of the note-takers was trained in participant observation. A key feature of deliberative stakeholder consultations is that the facilitator plays a minimal role once the debate starts [28, 29]. The purpose of a passive role of mediators was to identify implementation barriers that would exist in a real-world context where there are no researchers to provide explanations and health professionals can only use communication tools offered by PERSPECTIVE.

The small group deliberations began with a 10-min presentation by experts presenting background information on the risk stratification mode, followed by a 10-min open forum question period. At the close of the question period, the facilitator provided a set of questions that were to be addressed during the deliberative phase. The following questions were offered for discussion: (1) What do you think about the communication tools developed by the study? (2) Which communication tool will be the most useful to you? Which one will be the least useful? (3) In your opinion, what are the obstacles to the implementation of the new risk stratification model in clinical practice? What are your suggestions to overcome these obstacles? (4) In your opinion, what should be done to optimize the implementation of the suggested risk stratification model in clinical practice? These questions were presented as a way of initiating and framing the debate. Participants were allowed to determine the direction of the discussion with no interference from the facilitators. Facilitators only intervened to clarify information or to prompt discussion if the discussion has ceased for several minutes. Discussions lasted 1–1.5 h. After the small group consultations, summary documents were generated by the researchers and sent to the participants for ratification to ensure that all stakeholder viewpoints were considered in the formulation of the collective statements.

The mixed group deliberation included two participants from the small group deliberations: a family physician and a genetic counselor. The session followed the same format as the small group deliberations, with the exception that during the expert presentation the facilitators highlighted relevant themes and points of agreement and disagreement elicited from the previous small group deliberations that were offered for discussion. The participants were asked to address the following questions: (1) How should women access the screening program? Would this be different if the woman did not have a family physician? (2) When results are provided, whom should they be given to, and what should be the follow-up? (3) Where should this information be included in the communication tool(s)? A summary of a mixed session was sent to the participants for ratification.

All sessions were audio-recorded and transcribed. Inductive thematic content analysis was performed on the field notes and transcripts. Two coders (SP and JG) independently developed a coding scheme and analyzed the data. They discussed divergent codes until they were reconciled and re-analyzed the data. Two outputs of the deliberations comprised the conclusions reached by the deliberants (the deliberative outputs) and the themes that emerged throughout the discussion (analytic outputs) [30]. These outputs were translated into recommendations for improved communication tools (not reported here) and recommendations for an implementation strategy. The findings reported in this manuscript reflect the common themes and deliberative outputs arising from detailed discussions at each deliberative consultation.

Quality of deliberations was evaluated quantitatively, in terms of equality of participation, using counts of comments from each of the deliberants and instances of turn-taking [31]. All participants completed a survey to assess their perception of the quality of the session. The quality of the deliberation was also assessed qualitatively, using participant observation. One of the researchers examined the interpersonal dynamics and non-verbal cues, for evidence of specific deliberants exerting power over others that might impact their willingness or capacity to speak or refute others’ statements. The combination of qualitative and quantitative methods to measure the quality of deliberations has been proposed and validated by de Vries et al. [31]. This approach was demonstrated to be reliable in the estimation of deliberations quality, and it provides better insight in how the opinions are formed [31]. Quotes are used to illustrate the manner in which particular themes are discussed, as with content analyses of focus groups or group interviews. As deliberative stakeholder consultations involve an examination of deliberative outcomes in relation to the entire dynamic deliberative exchange [28], only a limited selection of quotes are provided.

## Results

### Qualitative data

Based on the results of deliberations, practical recommendations on the optimization of the communication tools (not reported) and on the implementation of the breast cancer risk stratification screening approach (Table 1) were developed. Our key findings, reflected in the main themes discussed during the deliberations, are presented below under the two main topics: implementation issues and assumed benefits.

**Table 1:** Main points of agreement in deliberations from health professionals for the implementation of PERSPECTIVE

Consider the harms and benefits of screening for women with low and high risk and the feasibility of tests for the general population. The decision to introduce the program needs to be based on strong evidence that benefits are greater than the harms.	
Provide justification of the value (cost-benefit) of population-wide screening.	
Ensure implementation of a centralized program that involves dedicated personnel to communicate tests results, provide psychosocial support, and organize proper referrals and follow-up for those who undergo screening.	
Ensure that all the recommended tests are covered by public system or health insurance.	
Define the concepts of “breast cancer diagnosis” and “risk for breast cancer” and clearly explain the difference between them.	
Integrate the calculator into EMRs and, if possible, have the information auto-populate the fields, with prompts for additional information.	
Prepare the socio-political environment and education support. Troubleshooting and constant feedback are required to improve the process.	

### Implementation of the program

#### Introduction of the program and access to screening

Time restriction was among the most important concerns that primary health care professionals (PCPs) voiced. They argued that introduction to the program by PCPs solely may not be feasible due to the lack of time at a typical appointment. Several ways to solve this problem and to optimize the implementation were discussed. PCPs suggested a stepwise access to implementation. The first step would be to use public campaigns. In their opinion, invitation perceived as being issued by the government would add to the chances of being accepted. The second step would be access to the program through PCPs. One of the suggestions was obtaining more evidence that the risk stratification model is beneficial for women. It was believed that this will encourage PCPs to use the program and provide justification of the value (cost-benefit) of population-wide genomic screening. Both groups of participants have highlighted that the decision to introduce the risk-based approach needs stronger evidence that benefits are more than harms. Another important issue was raised by genetic counselors. Participants of this group have expressed concern that if introduction and access are expected to be executed through PCPs exclusively, women without a family physician will be less informed and have no access to screening.

At the mixed session, it was agreed that an alternative way to advertise and introduce the risk-based approach (i.e., rather than through PCP offices), such as through a public advertising campaign, should be considered. Moreover, the deliberants suggested that, before granting women easy access to screening, the centralized psychosocial help program (possibly, with an engagement of a nurse or other dedicated personnel) should be implemented. Health professionals working in this program would help women understand the risks and benefits of participation in screening and explain screening results.

#### Communicating results of individual risk estimation

Optimal modes of communicating results of risk estimates to health professionals prompted a long discussion among genetic counselors. They suggested that the results could be posted on a website with access by health professionals. Alternatively, the results could be included in an electronic medical record (EMR) and health professionals would be able to view the results. If any breast cancer predisposition genes are to be tested, test results should be communicated to genetic counselors. At the mixed session, the participants agreed that results should be available at the same time to women, their physicians, and other clinicians involved in their screening and follow-up.

One of the main topics of discussion at both the small and mixed consultations was how to explain the results to women. PCPs were concerned that explaining risk and translating results would be very time-consuming. They suggested engaging a nurse and considering the creation of a helpful online tool and discussed these at length. As an example of a more effective way of explaining test results, the PCPs discussed the “absolute CVD risk/benefit” calculator, which uses a “hundred faces” diagram (e.g., iconoray) to explain a person’s estimated risk of suffering a heart attack.

I do this kind of thing quite regularly for cholesterol […] one of the most common questions I get is: ‘Doctor is my cholesterol normal?’ And I say to them, well I have a better question for you, ‘What is the chance that you will have a heart attack in the next ten years?’ And they look at me and they say, “Well what do you mean?” And I say that’s why we do the cholesterol test, to estimate what is the chance? And then I show them this 100-person iconoray that I have on my screen (Family Physician, Individual Deliberation).

The genetic counselors were concerned that without easy access to a health professional who can address questions in a timely fashion and explain the meaning of results, women may experience a lot of stress wondering what the results mean for their health and well-being. On the other hand, communicating results by PCPs exclusively would put those without a family doctor in a disadvantageous position, and communication of results by genetic counselors would lead to longer wait times. Notably, genetic counselors mentioned that many health professionals do not sufficiently understand “risk” and that the development of a comprehensive tool to help health professionals understand the meaning of results should be considered. Indeed, consultations with PCPs showed that the participants were unsure how to interpret the 10-year risk for breast cancer based on the communication tools provided by PERSPECTIVE and they were concerned about how to communicate the risks calculated by BOADICEA to women.

At the mixed session, participants agreed that the best way to communicate screening results is through a health professional who has a relationship of trust with the person. They felt that special attention should be paid to explaining all uncertainties in test results to avoid increasing women’s anxiety. Women who do not have family physician need to have access to an alternate health professional who can explain these results. Participants felt that engaging nurses or other dedicated personnel would be a good solution to the problem if the personnel was educated on the correct interpretation of the risks.

#### Perspectives on women’s decision making regarding participation in the program

PCPs highlighted the importance of involving women in making informed decisions to undergo screening for breast cancer risk stratification. In their opinion, women who are worried about their health due to personal reasons and those who have objective reasons to worry (e.g., family history of breast cancer) will be more willing to participate. Another reason why women may be willing to undergo screening is the perception of screening as an element of a periodic health exam. PCPs felt strongly that introducing the program will decrease anxiety in women and allow them to engage in shared decision making. This, in turn, is expected to reduce the inappropriate use of opportunistic screening.

#### Obstacles to using the model in a family physician’s office

Among the major obstacles to implementation acknowledged by both types of health providers was the lack of time for PCPs during a typical 20–25-min appointment. Many felt that there is simply not enough time to introduce the program, explain risks and benefits of participation, enter the data in BOADICEA, calculate the risks, and explain the test results. As mentioned above, one of the ways to overcome this obstacle suggested by both groups of health professionals was by engaging nurse or other trained personnel for some or all steps of this process. Moreover, if the program becomes a public health matter, then public health nurses could be involved. The use of EMR was mentioned as a means to help facilitate program implementation as the information (or at least part of the information) could be automatically populated from the woman’s medical chart. Finally, integration of the tool in the EMR was also suggested as helping to ensure the routine use of risk stratification tools by PCPs. The participants highlighted that health professionals involved in this process would need to be trained to use the BOADICEA model and other communication tools developed for PERSPECTIVE.

Participants believed that providing women with a visual chart accompanied with verbal explanations would help decrease anxiety level and avoid overestimation of risks. They noted that this would also make doctor-patient encounters less time-consuming and, therefore, increase the feasibility of the screening program. In this regard, the “absolute CVD risk/benefit” tool was again mentioned as an example of a helpful tool for communication with women. As an important step to implement the new screening program suggested by PCPs, pilot-testing was suggested to ensure the feasibility of using the BOADICEA model and communicating program-related issues to women in the context of the PCPs office.

Another important topic that emerged during the discussions was the matter of skepticism regarding the benefits of mammography screening among PCPs. The participants felt that this attitude may be an obstacle to implementation.

#### Referring women for follow-up

The deliberants discussed referrals for follow-up with regard to women who do not have a family physician. They suggested that, in this case, explanation of screening and communication of results could be performed by the trained nurse. They could also refer the women to either a family physician or a risk clinic and coordinate their testing. The best way, however, would be the development of a centralized program outside the PCP’s office with dedicated personnel to coordinate population-wide screening. This would ensure timely access to the risk clinics for women with intermediate and high risk, especially for those who have no family physician.

#### Correct interpretation of the program and its advantages

A major concern of PCPs was the interpretation of the meaning of the new breast cancer risk stratification approach and its advantages. One of the concerns raised by the group of genetic counselors and supported by PCPs was that of the perception of women of their risk group as assigned by BOADICEA. For example, women with a family history of breast cancer who have been assigned a population-level risk by the risk stratification model may have problems accepting this result. The deliberants from both groups suggested that online educational resources may help women understand risk estimation and reduce anxiety; however, participants felt that the help of an educated health professional could be essential to limit anxiety in these women.

#### Uncertainty about the difference between risk assessment and screening for disease

One of the genetic counselors mentioned that risk assessment and diagnosis need to be more clearly defined in the communication tools and that “breast cancer risk assessment” and “breast cancer diagnosis” are different concepts.

For me, dealing with risk assessment all the time, I think it’s perfectly adequate for me… With the concerns that you raised about what patients are going to think, I think we need to make sure that they say, this is not a diagnosis […] What I would like to see on this is more of a clear explanation of what will happen following the process (Genetic Counselor, Mixed Deliberation).

This need was observed in the deliberations with the PCPs. In their discussion, PCPs confused the assessment of breast cancer risk for women with communication of benefits and harm of participating in the recommended screening for disease reduction. For instance, the PCPs underscored the importance of helping women understand all the benefits and harms that this new assessment would bring to help make an informed decision. While discussing women’s decision making, they asserted that the possibility of over-diagnosis needs to be explained to women.

I know some women who have deliberately chosen not to go through screening umm and some of them are very bright women who know about over-diagnosis and who know about the harms that can come from screening and then made the conscious decision not to go. Because they actually see the harms as being greater than the benefits for themselves (Family Physician, Mixed Deliberation).

Moreover, in the small groups and during the mixed session, the PCP suggested providing women with online information to educate them on the risks and benefits of screening and help them make a decision regarding their participation in the program. PCP discussed the “absolute CVD risk/benefit” calculator as an example. These issues, however, are related to disease screening and not to the risk stratification. This confusion persisted throughout the small and mixed sessions.

### Benefits of the program

#### Benefits for health professionals

According to PCPs, being able to use a validated tool for guiding screening practices, rather than being influenced by women’s anxiety, would be beneficial.

I really like the idea of having some validated tool that I could use because otherwise we are just reacting emotionally to what is the unknown or the uncertainty of the future with no sense of probability. So having the tool gives you…what is the numerical chance of having an event and then you can sort of start thinking more rationally about whether or not it’s worth ‘opening Pandora’s box’ by going into a mammogram (Family Physician, Individual Deliberation).

Genetic counselors expressed their hope that the program would help other health professionals better understand whom to refer to them and how to refer*.* They expect that the referrals would become more appropriate, which would make their work more efficient.

#### Benefits for women

PCPs agreed that implementation of this new program could be beneficial for women.

People do not really know what their risk is. Some who are anxious may overestimate and others kind of deny everything (Family Physician, Individual Deliberation).

For women who tend to worry, and/or have their reasons to worry about having breast cancer, knowing their risk group would be reassuring. PCPs raised the question of whether cultural differences could determine differences in the magnitude of potential benefits. For example, they suggested that certain population groups (e.g., recent immigrants) may benefit less from the program. They may be less informed on the benefits of screening in general, and it may therefore be more difficult to explain the risk-based approach to them. It was suggested that the explanations for these groups of women could be done by the trained nurse, preferably one with an understanding of cultural preferences. Genetic counselors added that the risk stratification model and communication tools would be a good option to assess risk without enduring the 2-year wait period that currently exists for access to genetic counseling.

It’s like a perfect tool to assess risk, it would not really require a genetic consult and the two-year wait that goes with getting a genetic consult (Genetic Counseling, Individual Deliberation).

Women will be able to assess their risk without seeing a genetic counselor. Those of them assigned the near-population risk will not need counseling. As a result, shorter wait lists are expected, and those with medium and high risks will have more access to genetic counseling.

It was agreed at the mixed deliberation session that the program will most likely be beneficial for people whose risk level is higher than the population risk level. The participants remained uncertain about whether the benefits of the program are greater than the risks for people at the low (population-level) risk and if there is sufficient evidence to justify implementing a population-wide risk assessment. In their opinion, more research is needed to show how the program may affect people who are not at risk in order to convince PCPs to accept this risk-based approach. It was also highlighted that without proper training for health professionals, the risk-based screening will likely not work. Women may not truly benefit from easy access to the screening unless their health professionals understand the risks and benefits of screening and can properly interpret the results. This again reflected the PCPs’ confusion between risk assessment and disease screening.

### Quantitative analysis of quality metrics

Every participant completed the survey on the quality of the sessions (*n* = 13). The responses indicate that all participants felt that the group respected their opinions, that the facilitators listened to them, and that the process that led to the group response was fair (see Table 2). The results of the participant observation confirmed that the deliberative process was characterized by respectful debate and interest in others’ opinions. The number of turns taken and percentage of discussion (see Table 3) was fairly evenly distributed between groups, with the exception of one PCP and one genetic counselor who tended to dominate the conversation. Participants still felt the group response was fair, so this suggests that these individuals expressed views with which the others agreed. After framing the discussion, the facilitators’ engagement in dialog was minimal, as expected.

**Table 2:** Quality metrics by participant for deliberative outputs

Group	Words spoken^b^		Turns taken	
	Number	Percentage	Number	Percentage
Genetic counselors				
Facilitator(s)^a^	397	6	15	4
Participant 1	1210	17	102	29
Participant 2	3005	42	112	32
Participant 3	2123	30	118	34
Family physicians				
Facilitator(s)^a^	238	3	13	5
Participant 1	995	13	45	18
Participant 2	3015	39	107	42
Participant 3	3463	45	87	35
Family physicians				
Facilitator(s)^a^	439	7	20	14
Participant 1	222	4	4	3
Participant 2	580	10	15	10
Participant 3	967	16	29	20
Participant 4	1527	26	36	24
Participant 5	2142	36	43	29
Mixed session				
Facilitator(s)^a^	477	8	16	13
Participant 1	1606	28	48	38
Participant 2	3668	64	62	49

^a^Facilitators’ input; in each stakeholder deliberation, data from two facilitators was merged

^b^Some words inaudible or spoken collectively by more than one deliberant. Percentages are based on the total number of words spoken by participants

**Table 3:** Perceived quality of deliberations (*n* = 13)^a^, combined total for every group with mixed group representing two previous respondents

Question	Number satisfied (%)^a^
Do you feel that your opinions were respected by your group?	13 (100%)
Do you feel you were listened to by your facilitator?	13 (100%)
Do you feel that the process that led to your group’s response was fair?	13 (100%)
How willing are you to abide by the group’s final position, even if you personally have a different view?	10 (70%)
How helpful did you find question and answer interaction with the experts?	10 (70%)
How helpful did you find the formal presentations given by the experts?	10 (70%)
How helpful did you find discussing the issues with other participants?	13 (100%)
How much did attending the session change your *understanding* about the use of this new breast cancer screening approach?	11 (80%)
How much did attending the session change your *opinion* about the use of this new breast cancer screening approach?	10 (70%)

^a^Combined total for every group with mixed group representing two previous respondents.^b^Ranked on a scale of 1–10 with “1” very unsatisfied and “10” very satisfied. Any response ≥ 6 was counted as satisfied and < 6 as unsatisfied

## Discussion

Breast cancer screening is well established, with decades of observational and experimental evidence for its effectiveness [32]. Based on the results of large randomized controlled trials, breast cancer screening was shown to decrease mortality from breast cancer by an estimated 20% to 30% [9, 11, 33, 34]. Breast cancer-related mortality, however, remains one of the most important problems in Canada and worldwide, with increasing rates in developing countries [4]. The need for a more personalized approach to breast cancer screening with the development of conceptually new, multilevel models to coordinate the screening process has been highlighted previously [9, 32]. Onega et al. [32] called the existing screening system for breast cancer the “one-size-fits-all” approach, one that is not tailored to levels of risk and, therefore, that does not allow for the balance of the risk/benefit ratio for screening. Current guidelines are designed with this general approach and are not very helpful for clinicians who are most often concerned about what action to take for individual women [16]. In our study, we explored the perspectives of health professionals on the implementation of the new personalized risk-based screening approach, PERSPECTIVE, based on the calculation of individual risk for breast cancer development and subsequent alignment of women with different risk levels with the corresponding recommendations for disease screening.

Our study demonstrated that the introduction of the new step (assessment for risk stratification) adds complexity to the screening process and needs a different approach to implementation. One of our key findings was health professionals’ confusion between the two steps of the screening approach: risk stratification “screening,” which is actually an evaluation of the level of risk, and “screening” for disease. This occurred even when the PCPs were referring to visual aids from the expert presentation. The two dimensions of the screening approach introduce added complexity for health professionals in their explanation of the program’s meaning and benefits. This complexity may therefore require engagement of other health professionals (e.g., nurses or other essential personnel) in addition to PCPs. Moreover, it may require the creation of a centralized public health program with dedicated personnel who will help PCPs to introduce and maintain risk stratification and subsequent screening participation.

The confusion between the two steps of the screening approach was an important finding in our study. For example, the deliberants suggested creating an online educational tool that would help inform women about the risk and benefits of screening. They referred to the “absolute CVD risk/benefit” calculator [35] with the “hundred faces” iconoray as a tool that they use frequently and find easy to explain. This tool, however, does not aim to explain risk stratification. Likewise, recommendations on disease prevention (e.g., life-style factors and treatment options) included in the calculator are not based on an individually calculated percentage of risks. In contrast, the new personalized risk assessment approach offered by PERSPECTIVE aims to establish the individual level of risk and to tailor the subsequent recommendations for disease screening and prevention to the level of risk category for each individual woman. The confusion was also demonstrated in discussions about explaining the risk and benefits of participation in early (less than at 50 years) mammography screening for disease prevention, even though this matter is related to the second step of the screening approach: screening for disease. On this topic, over-diagnosis was also mentioned several times, even though over-diagnosis is an issue of screening-for-disease, not screening-for-risk programs. The PERSPECTIVE program seeks to provide recommended disease screening opportunities for women with intermediate and high risk. Women in the low-risk group would continue to undergo current standardized screening.

The confusion between the two steps of the proposed risk-based screening approach could mislead health professionals and women towards the wrong meaning of risk stratification and make them reluctant to encourage women to participate. To make the program implementation more feasible and to encourage the participation of women in new screening strategies based on individual risk, it is important to ensure adequate understanding of the multistep risk stratification screening by all. Health professionals should understand and be able to convey to women that the new approach offered by PERSPECTIVE is not meant to modify the existing screening program for women with near-population risk but rather provides women at a higher-than-population risk with additional, personalized measures. Since women with near-population risk will be assigned their risk level earlier, it will help decrease anxiety and avoid unnecessary referrals and procedures for those women from this group who are concerned about their breast cancer risk before the age of 50 due to personal reasons and would otherwise undergo the opportunistic screening. Training of all parties engaged in screening is essential to any screening program’s success [36], and insufficient training is one of the major barriers to successful implementation of screening programs [27, 37, 38]. As mentioned in the review of Rainey et al. [27], additional training in the communication of risk information is critical to increase women’s understanding. In line with these data, the importance of proper training and education of PCPs and other dedicated personnel was highlighted by health professionals as one of the key points of implementation. This could be done, for example, by the introduction of an online educational program, as suggested during the deliberations. It is important, however, to include training protocols in the original planning and design of the program’s implementation. In addition, creating a tool geared towards explaining the meaning of the different steps of the program and its benefits to women would be helpful. As reported by other researchers [36] and confirmed in our study, a major concern for PCPs performing such screening is time constraint. It is critical, therefore, to allocate nurses or other trained personnel to help physicians explain the meaning and benefits of the multistep program to the population.

Primary healthcare has always been a key to early detection of diseases as well as disease prevention [36]. PCPs, who are often women’s first contact in the healthcare system, play a fundamental role in implementing screening guidelines [39]. The breast screening participation rates in Canada were 44.3–49.3% for women aged 50–74 years in 2009–2012 [40]; however, these rates are expected to be lower among women without a family physician since in some provinces (e.g., Yukon, British Columbia), women must have a family physician in order to be eligible for screening [41]. On the contrary, as was mentioned during the deliberations, women with family physicians who have good relationships with their family doctors may be more willing to participate in the new screening program and may be more comfortable if their screening results and explanations regarding their risk groups were communicated by a health professional they trust. PCPs, however, are overloaded with their current essential work, creating time restrictions that may prevent many of them from participating in such screening programs [42, 43]. With the increasing complexity of screening systems and the introduction of tools and programs for personalized screening, new strategies need to be considered to introduce and support [44] these programs. Screening programs must be planned based on the needs and availability of primary care practices and the interests and experience of PCPs [36]. One of the suggestions of PCPs during our deliberations was to integrate the risk calculator into the EMR, so as to decrease the time needed to estimate the risks and ensure routine use of the tool in PCP’s offices. In addition, engaging nurses or other educated personnel in all steps of screening for breast cancer risk could compensate for the lack of time typically allotted to consultation with a family physician. Moreover, as the results of our deliberations suggested, use of a centralized public health program with engagement of dedicated personnel is critical to ensure effective screening. The program would help PCPs to introduce women to screening, provide psychosocial support to those who have difficulties with perception of their risk level, and coordinate proper screening and follow-up. Establishment of a centralized program outside of PCP’s offices would also ensure equity of access to the program by women who lack a family physician. Creating such a public health program, however, will not be possible without a clear understanding of risk and benefits of the new screening approach by everybody who is involved in screening, such as health professionals, policy makers, and women.

The identified barriers to the implementation are in line with the recent publication of Rainey et al. [27]. Our methods, however, brought another dimension to the exploration of implementation barriers: they allowed for interactions between relevant health care providers rather than surveying individuals. Our research highlights the issues that are expected to arise in real-world settings where PCPs would work together to implement the PERSPECTIVE program; our findings will help to inform a communication tool to better guide this process.

Despite the noted difficulties concerning implementation, health professionals acknowledged the substantial benefits of the proposed PERSPECTIVE program. PCPs embrace the opportunity to use validated tools to make decisions based on recommendations tailored to breast cancer screening risk groups. Genetic counselors hoped to see more women who really need to be counseled rather than unnecessary referrals. Agreement was reached on the fact that implementation of the new program would be beneficial for certain groups of women: women with intermediate and high risk. Women who worry about their health or, specifically, about their risk of developing breast cancer, will be reassured or will be given an opportunity to start screening for disease earlier. Health professionals, however, foresee problems that implementation of the risk-based approach may bring to other groups: those who have difficulty accepting the risk group to which they have been assigned or those who have problems understanding the meaning and the benefits of the program, such as recent immigrants. Based on their recommendations, we suggest creating a reliable centralized system outside of PCP’s offices to solve these problems by offering psychosocial help and to ensure access to trained nurses or other personnel for women, especially those without a family physician. The capacity to involve different health professionals or trained individuals should be evaluated by health system administrators and institutional administrators. The main topic of concern for health professionals was justification of program implementation as a population-wide screening, based on their uncertainty that it will be beneficial for women with near-population risk. In our opinion, however, this uncertainty was due in part to the general confusion between the two steps of “screening.” In our view, there is little harm in women of low-risk learning of their risk group within the PERSPECTIVE program. We acknowledge PCPs’ feasibility concerns, since the new population-wide screening would add a substantial amount of work to the routine of PCP’s practice [44]. This problem, however, could be solved by engagement of nurses or other essential personnel and by creating a centralized public program to assist PCPs in handling population-wide screening. As researchers, however, who are conveying the message of stakeholders on the proposed new screening program for breast cancer risk stratification, we need to highlight that one of the main recommendations of health professionals was to obtain more evidence on the prevalence of benefits over harms before its introduction in the general population [16].

While deliberative stakeholder consultations are intended to elicit stakeholder perspectives while minimizing researcher bias [28, 29], it is important to note that our study has some limitations. The typical sample size for deliberative stakeholder consultations is between 6 and 10 per small group with at least six in the mixed group. While our genetic stakeholder consultation group only had three participants, this actually represents 60% of all genetic counselors in the region (5 total excluding those currently on leaves of absence) and one of these participated in the mixed session. For the family physicians, our consultations took place during a particularly problematic primary care health reform that resulted in a number of participants canceling last-minute. The impact of our low numbers was mitigated, however, by two design features of the deliberative stakeholder consultations. First, the addition of participant observation permitted us to assess interpersonal dynamics, and non-verbal cues, and allowed us to conclude that participants were in agreement. This was also reflected in the results of the quantitative assessment and the surveys. Second, deliberative stakeholder consultations are designed to detect implementation issues that may not be documented in existing empirical literature. Despite the small numbers, our deliberations were successful in identifying the significant issue of confusing categorizing risk versus disease identification that is the objective of all current screening programs. Without this work, a concerted effort to clarify this issue for women, health care professionals, and policy makers may not have occurred. This would create a potentially significant implementation barrier for any risk stratification program. Deliberative stakeholder consultations are not designed to provide definitive evidence for implementation but to highlight potentially problematic areas for further investigation and we believe we succeeded in accomplishing this.

## Conclusion

Our study was the first to evaluate the perspectives of health professionals, including PCPs and genetic counselors, on the implementation and benefits of a new program for breast cancer risk stratification with the purpose of personalizing subsequent screening for disease by engaging health professionals in deliberations on this topic. The collective points of agreement of the health care professionals for the program implementation have been presented (Table 1), as well as points of contention. We have noted that this new approach to screening transforms breast cancer screening into a sophisticated multistep process. It does, however, require more clarity in communication with health professionals. Health professionals need to understand the difference between assessing for the risk level and screening for disease; that is that there are two steps that require different approaches. It is important for them to know and to be able to convey to women that the PERSPECTIVE risk stratification approach is not meant to modify any existing screening program but to add more opportunities for early detection and prevention of disease for women with higher-than-population risk. In addition, our deliberations showed that, with an increase in complexity of screening and the introduction of personalized screening programs, it may not be feasible to restrict coordination of screening to PCP offices. Engagement with other health professionals or additional personnel will assist all stages of the screening process. Moreover, a centralized public health program may need to be developed for implementation and for maintaining effective screening. This study provides perspectives on an important evolution of disease screening in primary care and public health.

## Abbreviations

BOADICEA: Breast and Ovarian Analysis of Disease Incidence and Carrier Estimation Algorithm
EMR: Electronic medical records
PCPs: Primary health care professionals
PERSPECTIVE: Personalized Risk Stratification for Prevention and Early Detection of Breast Cancer
